# Hepatic, adipocyte, enteric and pancreatic hormones: response to dietary macronutrient composition and relationship with metabolism

**DOI:** 10.1186/s12986-017-0198-y

**Published:** 2017-07-05

**Authors:** Bridget M. Hron, Cara B. Ebbeling, Henry A. Feldman, David S. Ludwig

**Affiliations:** 1 Division of Gastroenterology, Hepatology and Nutrition, Boston Children’s Hospital, 300 Longwood Ave., HUN Ground, Boston, MA USA; 2 New Balance Foundation Obesity Prevention Center and Division of Endocrinology, Boston Children’s Hospital, 300 Longwood Ave, Boston, MA USA; 3 Clinical Research Center, Boston Children’s Hospital, 300 Longwood Ave, Boston, MA USA

**Keywords:** Insulin sensitivity, Energy expenditure, FGF-21, Metabolic hormones, Dietary composition

## Abstract

**Background:**

We sought to characterize the effects of dietary macronutrient composition on various hormones implicated in the regulation of insulin sensitivity (IS) and energy expenditure (EE).

**Methods:**

Following 10–15% weight loss, 21 overweight subjects consumed 3 weight-loss maintenance diets [low fat (LF), low glycemic index (LGI) and very low carbohydrate (VLC)] in random order, each for 4 weeks. At baseline and at the end of each treatment period, fasting samples for fibroblast growth factor (FGF)-21, heme-oxygenase-1 (HO-1), chemerin, irisin, secreted frizzle-related protein (SFRP-4), total bile acids, ghrelin, gastrin inhibitory peptide (GIP), peptide-Y, and amylin; hepatic and peripheral IS; and EE were obtained. Analyses were controlled for age, gender, baseline body mass index, and diet sequence.

**Results:**

FGF-21 decreased (*P* < 0.0001), with differential effect by macronutrient composition (mean change from baseline ± SEM: LF −49.4 ± 16.6, LGI -58.6 ± 16.3, VLC -76.7 ± 18.2 pg/mL, *P* = 0.0002). Change in FGF-21 was inversely associated with change in hepatic IS [Beta = −0.565 units/log(ng/mL), *P* = 0.02], but not with peripheral IS or EE. Heme-oxygenase-1 (HO-1) increased (*P* = 0.003), without differential effect by macronutrient composition (LF 0.40 ± 0.26, LGI 0.98 ± 0.63, VLC 0.49 ± 0.29 ng/mL, *P* = 0.07). Ghrelin increased (*P* = 0.0003), while chemerin decreased (*P* = 0.001) without macronutrient effect. Total bile acid, irisin, SFRP-4, GIP, peptide-Y and amylin levels did not change.

**Conclusions:**

FGF-21 levels decreased with dietary intervention in proportion to carbohydrate content, and correlated with hepatic insulin sensitivity, suggesting a pattern of improving FGF-21 resistance. HO-1 increased in response to dietary intervention, a tendency to greater increase in response to the LGI diet. Dietary intervention affected ghrelin and chemerin, independent of macronutrient composition. These findings may elucidate relationships between dietary composition, insulin sensitivity and metabolism.

**Trial registration:**

NCT00315354.

## Background

Previous data suggest that dietary composition modulates weight-loss induced changes in energy expenditure, insulin sensitivity and satiety. For instance, among subjects following a feeding protocol, energy expenditure decreased by approximately 325 kcal/day more following a low fat vs. low carb diet controlled for energy intake [1]. Other studies have reported metabolic benefits and improved satiety with reduction of glycemic load [2, 3], but the mechanisms mediating these effects remain incompletely described. Hormones secreted from the gastrointestinal tract and related organs have been implicated in these relationships.

Fibroblast growth factor (FGF)-21, secreted by the liver, enhances glucose uptake into adipocytes [4]. Administration of FGF-21 to humans modestly decreases body weight [5], and in animal models improves insulin sensitivity, energy expenditure and hepatic steatosis [6], with maximal effect at low doses. However, FGF-21 levels are elevated in humans with obesity and type 2 diabetes mellitus [7], in association with markers of metabolic syndrome, which suggests a pattern of FGF-21 resistance [8] that may comprise a fourth component of the obesity-related resistance triad of insulin, leptin and sympathetic tone. Indeed, FGF-21 levels decrease with bariatric surgery [9], although the effect of diet induced weight loss has not been well-characterized.

Chemerin, a novel hepatocyte and adipocyte-derived hormone, is a chemoattractant for tissue injury that regulates adipocyte differentiation and thermogenesis. Chemerin levels are increased in obesity [10] and correlate with degree of fatty liver [11]. Chemerin levels decrease with weight loss from diet [12] and bariatric surgery [11] but effects of dietary macronutrient composition are unknown. Bile acids, also produced by hepatocytes, modulate body weight, insulin sensitivity and energy expenditure in rodent models [13], though little is known about the effects of weight loss or dietary composition in humans. Heme oxygenase (HO)-1 is secreted by adipose tissue and reduces oxidative stress. Induction in animals decreases body weight, inflammatory cytokine profiles, and insulin resistance [14]. Levels are elevated in patients with type 2 diabetes [15], but the effects of weight loss and dietary composition on HO-1 are unknown.

Several novel proteins have recently emerged as potential mediators of insulin dynamics and energy expenditure. Secreted frizzle-related protein-4 (SFRP-4) is a newly discovered pancreatic protein that decreases insulin secretion [16]. Levels are elevated in subjects with type 2 diabetes, and appear to precede the development of type 2 diabetes [16]. The myokine irisin stimulates uncoupling protein-1 expression in adipocytes and prevents diet-induced obesity and diabetes in mouse models [17]. Serum irisin levels are lower in patients with obesity and hepatic steatosis [18], and increase with exercise [17]. The effects of diet-induced weight loss in adults and dietary composition on SFRP-4 and irisin levels are not well-characterized.

Multiple enteric and pancreatic hormones have emerged as important mediators of food intake. Ghrelin, secreted from the stomach, rises acutely with fasting to stimulate hunger, and increases with weight loss [19]. Peptide-YY (PYY), secreted by the distal ileum, increases with nutritional intake to convey a satiety signal. PYY levels decrease with diet-induced weight loss [19]. Amylin, which is co-secreted by the pancreas with insulin, decreases food intake and body weight [20], and levels decrease in response to weight-loss maintenance [19]. Taken together, this pattern of hormonal alterations can contribute to weight regain [19].

Gastric inhibitory polypeptide (GIP) is secreted by K cells in the duodenum in response to intraluminal glucose and fat. Mice lacking GIP receptor are protected against diet-induced obesity, due to higher energy expenditure, and insulin resistance [21]. In the setting of weight maintenance, GIP levels tend to be lower in response to low glycemic index diets [22]. The effects of weight loss on GIP levels are less clear.

In this study, we aimed to characterize the changes in these metabolic hormones in response to weight loss and diets varying in composition, and analyze the potential contribution of these hormones to changes in physiology and metabolism.

## Methods

### Study design

**Fig. 1:**
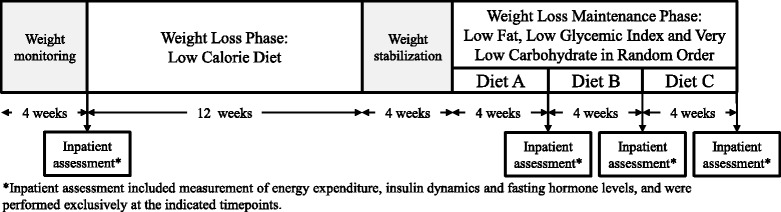
Study schema

The overall study design was previously reported [1]. In brief, participants who were overweight or obese, and otherwise healthy, achieved 10–15% weight-loss over 12 weeks by consuming a standardized reduced-calorie diet providing 60% of estimated needs and consisting of 45% carbohydrate, 30% fat and 25% protein. Participants then received each of 3 weight loss maintenance test diets for 4 weeks in random order (Figure 1). The diets were isocaloric, designed to achieve 100% of estimated caloric needs, but varied in macronutrient composition as follows: low fat (60% carbohydrate, 20% fat and 20% protein), low glycemic index (40% carbohydrate, 40% fat and 20% protein), and very low carbohydrate (10% carbohydrate, 60% fat, 30% protein). All study meals were designed and prepared in the metabolic kitchen at the Brigham and Women’s Hospital (BWH). Participants consumed one meal each weekday on site, with remainder of meals and snacks packaged for consumption at home, and completed a daily compliance diary. Participants were admitted to BWH or Boston Children’s Hospital for a four-day hospitalization at baseline (prior to weight loss) and after each of the three test diets (during period of weight maintenance). At each study visit, a full assessment was performed, including resting energy expenditure, insulin sensitivity and measurement of metabolic hormones. As previously reported [1], resting energy expenditure was measured by indirect calorimetry and total energy expenditure was measured under free living conditions by doubly-labeled water technique. Hepatic and peripheral insulin sensitivity were calculated from glucose and insulin responses to oral glucose tolerance test (OGTT) as per Abdul-Ghani et al. [23]. For hepatic insulin sensitivity, we calculated the product of the glucose and insulin areas under the curve during the first 30 min of the OGTT and then obtained the inverse. For peripheral insulin sensitivity, we calculated the rate of decline of glucose levels from peak to nadir during the OGTT and then divided by mean insulin levels. Participants maintained their habitual levels of physical activity throughout the course of the study. [1] The study was approved by the Boston Children’s Hospital and Partner’s Institutional Review Boards, and registered on clinicaltrials.gov (NCT00315354).

### Serologic hormone assays

Fasting samples were obtained at baseline (prior to weight loss) and at the end of each dietary treatment period (during weight loss maintenance) after a 12-h overnight fast. Serum was frozen at −80 °C until analysis. Samples from all time-points were available from 21 patients, except for HO-1 (*N* = 16) and irisin (*N* = 18).

FGF-21 [intra-assay coefficient of variation (CV) 1.9–5%, inter-assay CV 1.2–9%, limit of detection 15.6 pg/mL; Millipore, St. Charles, MO], chemerin (intra-assay CV 5%, inter-assay CV 4–6%, limit of detection 1.56 ng/mL; Millipore), heme oxygenase-1 (intra-assay CV < 10%, inter-assay CV < 12%, limit of detection 0.25 ng/mL; USCN Life Science Inc., Wuhan, Hubei, China), total serum bile acids (intra-assay CV <10%, inter-assay CV <10%, limit of detection 7.48 μmol/L; Crystal Chem, Inc., Downers Grove, IL), SFRP-4 (intra-assay CV <10%, inter-assay CV <12%, limit of detection 42.026 pg/ml; USCN Life Science Inc.) and irisin (intra-assay CV < 10%, inter-assay CV < 10%, limit of detection 4.15 ng/mL; Phoenix Pharmaceuticals, Burlingame, CA) levels were was measured by ELISA. Ghrelin (intra-assay CV 3%, inter-assay CV 7%, limit of detection 14 pg/mL), GIP (intra-assay CV 4%, inter-assay CV 10%, limit of detection3 pg/mL), active amylin (intra-assay CV 3%, inter-assay CV 21%, limit of detection 46.07 pg/mL) were analyzed by Luminex xMAP (Milliplex, Millipore, MA). Analyses were performed at the Joslin Diabetes Center Specialized Assay Core (Boston, MA). In a subset of 8 patients, as previously reported [24], fasting non-esterified free fatty acid (FFA) concentration was measured at Boston Children’s Hospital Clinical Lab by an in vitro enzymatic colorimetric assay, using Roche P Modular system (Roche Diagnostics, Indianapolis, IN) and reagents from Wako Chemicals USA (Richmond, VA).

### Statistical analyses

Effect of dietary composition on change in fasting hormone levels were evaluated by linear mixed effects repeated measures model with age, gender, change in BMI and diet sequence as covariates. Effect of weight loss was analyzed within a similar model by comparing the observed change to the null hypothesis of no change, using adjusted average change over each of the three test diet periods, and adjusting for age, gender, baseline BMI and diet and diet sequence as covariates. For those hormones which changed in response to dietary composition, the relationships with changes in insulin sensitivity and energy expenditure were analyzed using a similar mixed effects model.

Results are presented as mean ± standard error of the mean unless otherwise indicated. Hormones with skewed distribution were log_10_-transformed for analysis, and are presented as raw data mean (interquartile range) for generalizability. Statistical significance was set as a two-sided alpha value of *P* < 0.05. Using a strict Bonferroni correction for the 22 analyses performed, the inferences regarding the primary hormone of interest (FGF-21) are unchanged.

## Results

### Study population

Participant demographics, as previously reported [1, 25], and baseline characteristics are presented in Table 1 **.** Participants were 30.3 ± 5.7 (SD) years old, 62% male, with BMI 34.4 ± 4.9 (SD) kg/m^2^. Samples were available for all 21 participants who completed the study. FFA concentration was measured in a subset of 8 participants who were largely representative of the primary cohort [mean age 30.8 ± 6.4 (SD) years old, 50% male, with BMI 33.4 ± 4.8 (SD) kg/m^2^] [24]. All participants lost 10–15% of body weight during the initial weight loss period, and no subject regained more than 2% of this initial weight loss, or lost more than an additional 4%, during the weight maintenance period. Mean BMI decreased to 30.2 ± 0.9 kg/m^2^ following weight loss period, and stabilized during the weight maintenance period (low fat 29.8 ± 0.9, low glycemic index 29.7 ± 0.9 and very low carbohydrate 29.8 ± 0.9 kg/m^2^, *P* = 0.29).

**Table 1:** Patient demographics and baseline characteristics

Demographics	
Age (y)	30.3 ± 5.7
Gender	
Male	13 (62%)
Female	8 (38%)
Race	
White	4 (19%)
Black	8 (38%)
Asian	4 (19%)
Other	5 (24%)
Hispanic Ethnicity	4 (19%)
BMI (kg/m^2^)	34.4 ± 4.9
Insulin dynamics	
Fasting insulin (mIU/L)	11.4 ± 6.5
Peripheral insulin sensitivity^a^	0.53 ± 0.64
Hepatic insulin sensitivity^a^	0.82 ± 0.57
HOMA-IR	2.57 ± 1.55
Energy expenditure	
Total energy expenditure (kcal/d)	3248 ± 762
Resting energy expenditure (kcal/d)	1784 ± 376

Results reported as N (%) or mean ± standard deviation

Subset of data previously reported in Ebbeling et al. [1] and Hron et al. [25]

^a^Units calculated per Abdul-Ghani et al. [23]

### Effects of dietary macronutrient composition on hormone levels

FGF-21 decreased in response to dietary intervention (*P* < 0.0001), with differential effect by macronutrient composition (change from baseline: low fat −49.4 ± 16.6, low glycemic index −58.6 ± 16.3, very low carbohydrate −76.7 ± 18.2 pg/mL, *P* = 0.0002) as shown in Table 2. Insulin decreased with the intervention (*P* < 0.0001), also with differential effect by diet [low fat −5.3 ± 1.1, low glycemic index −6.0 ± 1.1, very low carbohydrate −6.1 ± 1.0mIU/L, *P* = 0.004, Table 2). HO-1 increased (*P* = 0.02), with a trend for a differential effect by diet (low fat 0.40 ± 0.26, low glycemic load 0.98 ± 0.63, and very low carbohydrate 0.49 ± 0.29 ng/mL, *P* = 0.07, *N* = 16, Table 2). Chemerin decreased (*P* = 0.001) and ghrelin increased (*P* = 0.0003) without differential diet effect. Total bile acid levels, irisin, SFRP-4, GIP, PYY and amylin did not change in response to the test diets (Table 2).

**Table 2:** Effects of weight loss and dietary composition during weight loss maintenance on fasting hormone levels*

	Pre-Weight Loss Baseline (*N* = 21)	Weight Loss Maintenance			*P*-value for overall diet intervention effect*	*P*-value for differential effect of diet**
		Low Fat (*N* = 21)	Low Glycemic Index (*N* = 21)	Very Low Carbohydrate (*N* = 21)		
Hepatic and/or adipose hormones						
FGF-21 (pg/mL)^a^	94.1 ± 18.0	44.8 ± 6.1	35.5 ± 7.8	17.5 ± 3.0	<0.0001	0.0002
Change from baseline		-49.4 ± 16.6	-58.6 ± 16.3	-76.7 ± 18.2		
Chemerin (ng/mL)	54.4 ± 3.4	46.6 ± 3.5	44.8 ± 3.0	45.9 ± 2.7	0.001	0.66
Change from baseline		−7.9 ± 2.1	−9.7 ± 2.1	−8.5 ± 2.4		
Heme-oxygenase 1 (ng/mL)^a^	0.69 ± 0.21	1.09 ± 0.44	1.66 ± 0.82	1.18 ± 0.47	0.02	0.07
Change from baseline		0.40 ± 0.26	0.98 ± 0.63	0.49 ± 0.29		
Total bile acids (umol/L)	10.7 ± 1.6	10.1 ± 1.4	9.4 ± 0.9	11.2 ± 1.9	0.89	0.45
Change from baseline		−0.6 ± 1.3	−1.3 ± 1.3	0.5 ± 1.7		
Pancreatic hormones						
Insulin (mIU/L)^a^	11.4 ± 1.4	6.1 ± 0.5	5.4 ± 0.4	5.2 ± 0.6	<0.0001	0.004
Change from baseline		−5.3 ± 1.1	−6.0 ± 1.1	−6.1 ± 1.0		
Amylin (pg/mL)	172 ± 50	179 ± 48	153 ± 49	164 ± 50	0.77	0.49
Change from baseline		6 ± 24	−20 ± 30	−9 ± 29		
SFRP-4 (pg/mL)	1086 ± 167	1307 ± 181	1346 ± 211	1220 ± 205	0.24	0.74
Change from baseline		196 ± 190	236 ± 208	110 ± 188		
Enteric hormones						
Ghrelin (pg/mL)^a^	11.5 ± 0.9	18.6 ± 2.9	18.8 ± 3.7	21.0 ± 2.9	0.0003	0.25
Change from baseline		7.1 ± 2.9	7.3 ± 3.5	9.5 ± 2.9		
GIP (pg/mL)	12.8 ± 1.7	14.4 ± 2.1	13.6 ± 3.1	13.2 ± 2.7	0.30	0.90
Change from baseline		1.6 ± 1.4	0.9 ± 2.4	0.5 ± 2.0		
PYY (pg/mL)^a^	46.1 ± 10.5	39.5 ± 4.5	36.1 ± 4.2	45.7 ± 10.2	0.75	0.33
Change from baseline		−6.6 ± 8.1	−10.0 ± 7.3	−0.4 ± 4.1		
Muscular hormones						
Irisin (ng/mL)^a^	127.5 ± 38.5	96.4 ± 18.1	90.0 ± 16.5	98.3 ± 15.9	0.21	0.23
Change from baseline		−31.0 ± 29.4	—37.5 ± 30.2	−29.2 ± 31.1		

Results reported as mean ± SEM. *N* = 21 for all variables except for HO-1 (*N* = 16) and irisin (*N* = 18)

*P-value tests the hypothesis that the average of the three changes is zero. Analyses adjusted for age, gender, baseline BMI, diet composition and diet sequence

**Analyses adjusted for age, gender, change in BMI, diet composition and diet sequence

^a^Results analyzed as log-transformed data

### Relationships between changes in metabolic hormones, insulin sensitivity and energy expenditure

**Fig. 2:**
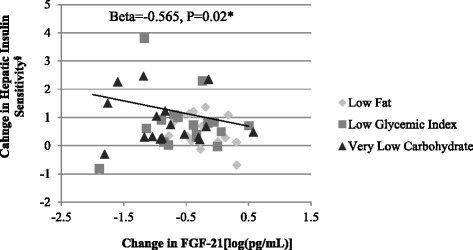
Relationship between change in FGF-21 and change in hepatic insulin sensitivity during weight loss maintenance, adjusted for age, baseline BMI, gender, diet composition and diet sequence. Hepatic IS units calculated per Abdul-Ghani et al. [23]

Effects of test diets on absolute changes in insulin sensitivity and energy expenditure were previously reported [1, 25]. A greater decrease in FGF-21 levels [β = −0.565 unit/log(ng/mL), *P* = 0.02, Fig. 2] was associated with greater improvements in hepatic, but not peripheral, insulin sensitivity. In the subset of patients in whom free fatty acid levels were measured, the relationship between changes in FGF-21 and hepatic insulin sensitivity was independent of FFA [β = −1.23unit/log(ng/mL), *P* = 0.04]. Changes in insulin and HO-1 did not predict changes in insulin sensitivity indices or changes in total or resting energy expenditure.

## Discussion

The main finding from this study is that FGF-21 levels decreased in response to dietary intervention with marked effect by macronutrient composition. Owing to differences in assay type, the absolute values of FGF-21 in our study are lower than some published reports [26], yet consistent with more recent publications [27, 28]. The greatest reduction in FGF-21 occurred on the very low carbohydrate diet. This finding extends recent observations that consumption of carbohydrate, specifically sugar, increases FGF-21 levels [29–32] and affects preference for sweets [30, 32], and that changes in sweet preference occur with sugar restriction. While the exact mechanism by which FGF-21 responds to nutrient intake is unclear, FGF-21 is stimulated by FFA [33], insulin [33] and ketones [34], and is necessary for changes in lipid and glucose metabolism induced by very low carbohydrate diets [35]. In our study, the effect of diet on FGF-21 was independent of FFA concentration, suggesting an alternate mechanism.

We speculate (but cannot prove) that the decrease in FGF-21 increased FGF-21 sensitivity, as evidenced by the close relationship with other physiologically relevant variables. Decreases in FGF-21 levels correlated with improvements in hepatic insulin sensitivity, consistent with previous studies showing that FGF-21 administration improves hepatic insulin sensitivity [34]. In this context, our findings suggest that obesity comprises a state of FGF-21 resistance. This hypothesis is in accordance with recently published data suggesting decreased FGF-21 with weight loss in an adolescent population [36]. In addition, the results raise concern for the proposed use of FGF-21 as pharmacologic treatment in obesity until physiological actions are further clarified [35]. This scenario may be analogous to leptin treatment in obesity and insulin treatment in type 2 diabetes, in which endogenous hormone resistance may limit efficacy and result in side effects.

We also measured several other novel obesity-related hormones. Our data are the first to demonstrate that circulating heme oxygenase-1 levels increase with dietary intervention, which may be an endogenous anti-inflammatory response to reduce oxidative stress and inflammation [14], though the clinical significance remains unclear. Though not statistically significant, the largest improvement in HO-1 levels was seen in response to the low glycemic index diet. While there is very little data regarding nutritional regulation of HO-1, this effect may have arisen as a direct result of macronutrient composition on HO-1 or indirectly via weight-loss induced improvement in insulin action [37]. We show that chemerin, a chemoattractant for tissue injury, decreased with dietary intervention, similar to previous reports [12]. The improved cytokine profile is most likely a response to weight loss, but may also reflect improved dietary quality. We also measured the novel hormones SFRP-4, a biochemical harbinger of type 2 diabetes [16], and irisin, the myokine that may mediate beneficial effects of exercise [17], and bile acids, implicated in regulation of energy expenditure [13]. These did not change throughout the study.

Consistent with previous data [19], fasting ghrelin levels decreased after dietary intervention without specific effect of dietary composition. While very low carbohydrate diets are typically associated with improved satiety and decreased post-prandial intake [3], ghrelin levels did not change across the test diets, likely the result of the complex regulation of hunger. In contrast to previous data [19], we did not observe effects of dietary intervention on the satiety hormones PYY or amylin.

The primary limitation to these analyses is that hormone concentrations were not measured immediately following the weight loss period. In addition, multiple comparisons may increase the false discovery rate. Nevertheless, our primary findings regarding FGF-21 would withstand adjustment for multiple comparisons. Dietary adherence was measured using a daily diary as completed by the participant. The clear differentiation in several metabolic variables with known dietary effects, such as triglycerides [previously discussed by Ebbeling et al. [1]], suggests excellent compliance. Strengths include a rigorously conducted feeding protocol, outstanding retention rate, and a crossover design allowing for maximal power and control for unmeasured confounding variables.

## Conclusions

In summary, FGF-21 levels decreased in response to dietary interventions varying in macronutrient content, likely secondary to decreasing carbohydrate content, and correlated with hepatic insulin sensitivity. Overall, this pattern is suggestive of improving FGF-21 resistance, independent of diet-induced effects on free fatty acid concentration. Dietary intervention affected HO-1 and chemerin concentrations, consistent with an improved inflammatory cytokine profile. Future studies are needed to further define the mechanisms underlying diet and weight loss effects on metabolic hormones.

## Abbreviations

BMI: Body mass index
CV: Coefficient of variation
FFA: Free fatty acid
FGF-21: Fibroblast growth factor-21
GIP: Gastric inhibitory polypeptide
HO: Heme oxygenase-1
LF: Low fat
LGI: Low glycemic index
PYY: Peptide-YY
SFRP-4: Secreted frizzle-related protein-4
VLC: Very low carbohydrate
